# TOOLS FROM OUR APPLIED GERONTOLOGY TOOLBOX: FLIPPING MINDSETS AND PROPELLING INNOVATION

**DOI:** 10.1093/geroni/igz038.899

**Published:** 2019-11-08

**Authors:** Roger A Anunsen, Roger Anunsen, Jan Abushakrah

**Affiliations:** Portland Community College, Portland, Oregon, United States

## Abstract

PCC’s associate degree program has long embraced an applied approach to gerontology education through its career pathway model. This model incorporates a rock-solid theoretical base, experiential learning, and skill-development, all grounded in the foundational, interactional and contextual AGHE Gerontology Competencies. The Competencies infuse all our courses, our five certificates and our degree to effectively prepare graduates for their next step, whether a transfer a specialized career. Our toolbox includes robust, wrap-around supports that include empowering advising and career planning, intentional internships, mentoring, networking opportunities, and our newest tool, The Flipping Mindsets Project. We will demonstrate how these evidence-based approaches provide students the knowledge, skills and confidence propelled by a positive mindset, to go boldly into any field of aging as innovators within existing jobs and programs or as ground-breaking entrepreneurs.

